# Implications of COVID-19 prevention on the occurrence of childhood diarrhea in the Semen Bench district, Bench Sheko zone, southwestern Ethiopia

**DOI:** 10.3389/fpubh.2024.1379232

**Published:** 2024-05-28

**Authors:** Bezuayehu Alemayehu, Seblework Mekonen, Argaw Ambleu

**Affiliations:** ^1^Department of Public Health, Mizan Tepi University, Mizan Teferi, Ethiopia; ^2^Water and Public Health, Ethiopian Institute of Water Resources, Addis Ababa, Ethiopia

**Keywords:** association, COVID-19, childhood, diarrhea, knowledge, practice, prevention, implications

## Abstract

**Background:**

Coronavirus (COVID-19) is a virus that occurred in Wuhan, China, in December 2019 and has spread to several countries. Although interventions in water, sanitation, and hygiene (WASH) for COVID-19 are likely a pre-existing response to childhood diarrhea, evidence of the effects of COVID-19 preventative strategies on childhood diarrhea has been lacking. This study aimed to assess the implications of COVID-19 prevention for the occurrence of childhood diarrhea in rural communities of Ethiopia.

**Methods:**

A community-based cross-sectional study was conducted from 10 May 2020 to 30 July 2020 involving selected households in the Semen Bench district, Bench Sheko zone, southwestern Ethiopia. A single population proportion formula was used to obtain a total of 768 sample sizes. Data were collected from selected households using a simple random sampling technique. Epidata 3.1 was used to enter the data and then exported to Stata 14 for analysis. Descriptive statistics along with binary and multivariable logistic regression analyses were used to identify factors of COVID-19 knowledge and practices related to childhood diarrhea. The chi-squared test was used to check the association between COVID-19 prevention and childhood diarrhea reduction.

**Results:**

A total of 720 (93.75%) households participated in the study to achieve the study objectives. Approximately 55% of the participants had a good understanding of COVID-19 prevention, while only 48.5% had good COVID-19 prevention practices. The prevalence of childhood diarrhea was 19.3% which was more common among households with poor practices of COVID-19 prevention. The respondents with poor COVID-19 prevention knowledge were 42% (AOR = 0.58, 95% CI: 0.398, 0.847, *P* = 0.005) less likely to develop childhood diarrhea than those who had good COVID-19 prevention knowledge. Households with poor practices for COVID-19 prevention were 75.1% more likely to develop childhood diarrhea than those who had good preventive practices for COVID-19 prevention (AOR = 1.751, 95% CI: 1.193, 2.571, *P* = 0.004). The lower risk of childhood diarrhea is significantly related to good COVID-19 prevention practices. However, households with no formal education and a lack of WASH facilities have a higher likelihood of having childhood diarrhea in the household.

**Conclusion:**

COVID-19 preventative strategies help reduce the prevalence of diarrhea in children. More research using prospective study designs and advanced statistical models is needed to better understand the implication of COVID-19 preventative efforts in reducing childhood diarrhea.

## Introduction

Coronavirus 2019 (COVID-19) is a globally burdensome virus transmitted through unhygienic handshakes, direct breathing, coughing, or sneezing from a COVID-19-infected person. The first case was identified on 13 March 2020 in Addis Ababa, Ethiopia. The WHO suggests handwashing with soap or using alcohol-based hand sanitizers, wearing a face mask, maintaining physical distancing, and staying at home if possible to prevent the spread of the pandemic (1).

The government of Ethiopia has implemented various non-pharmaceutical interventions (NPIs) to control the transmission of the virus. These measures include case identification, contact tracing, isolation, and quarantine, as well as promoting physical distancing and sanitary measures. In addition, the government has temporarily closed many social institutions, including all academic institutions and religious organizations. Additionally it has imposed restrictions on cross-country and inter-city public transport systems, and postponed the national election (2, 3) to curb the pandemic.

Handwashing with soap is a proven intervention method for reducing 45–55% of childhood diarrhea episodes, and it also serves as a major COVID-19 intervention (4). However, the findings revealed that handwashing with soap was not widely practiced in rural areas before the COVID-19 pandemic (5). This understanding of the pandemic's response has a substantial impact on reducing childhood diarrhea. Authors from eastern Ethiopia have reported that there is a reduction in childhood diarrhea among families which implement COVID-19 prevention knowledge and practices (6). Due to limited evidence regarding the implication of COVID-19 prevention practices and the occurrence of childhood diarrhea in rural communities of Ethiopia, it is crucial to conduct this study. Therefore, this study aimed at investigating the implication of COVID-19 preventative practices on the occurrence of childhood diarrhea. This research plays a crucial role in reducing the prevalence of childhood diarrhea and providing valuable information to decision-makers and researchers for generating hypotheses.

## Materials and methods

### Study area and period

**Figure 1:**
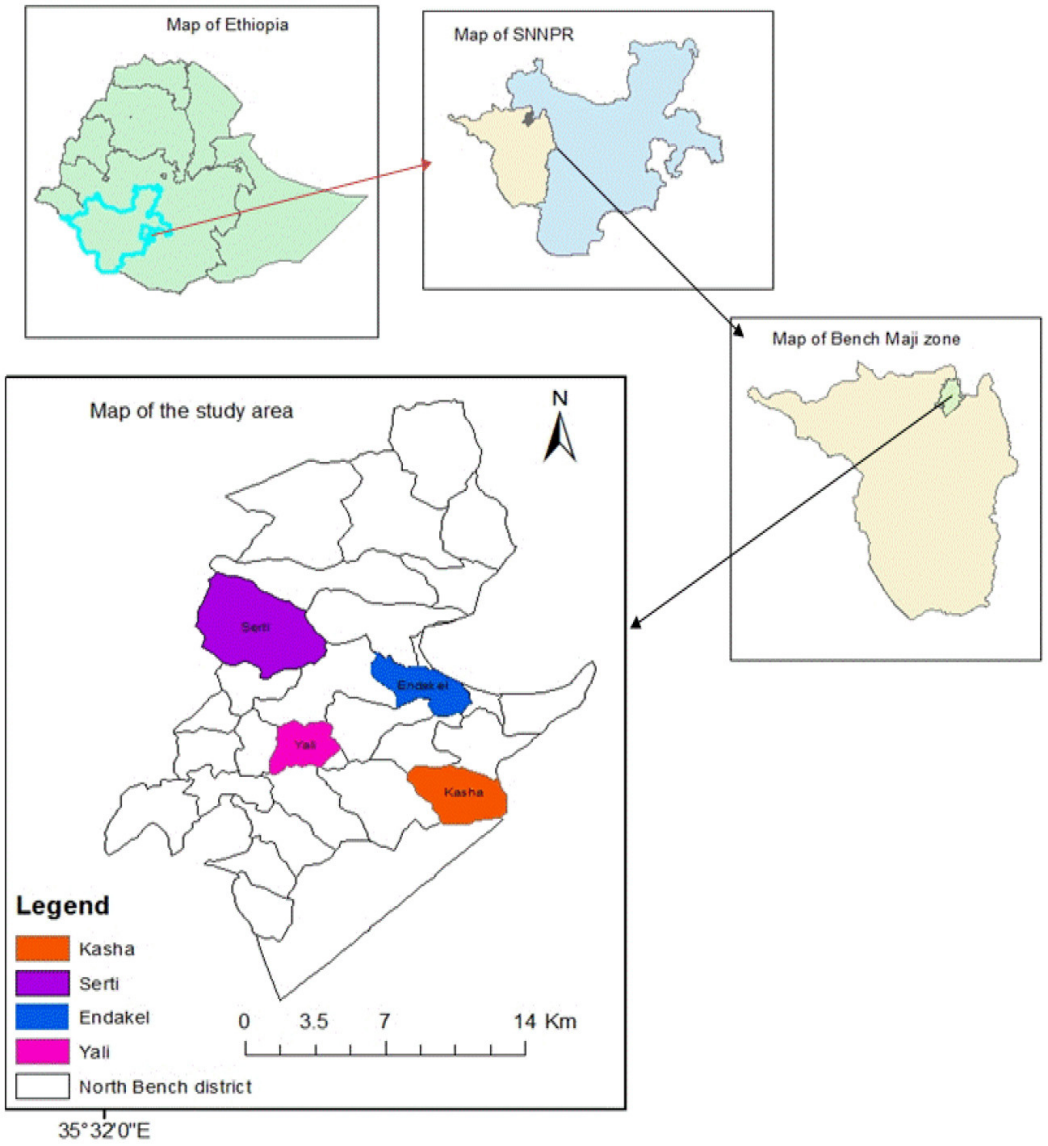
Map of the study areas. Esri reserves the right to grant permission for any other use of the image.

This study was conducted in the Semen Bench district, Bench Sheko zone, southwestern Ethiopia from 10 May 2020 to 30 July 2020. It is located 550 km from Addis Ababa, Ethiopia. The total population in the study area was 148,285, including 71,177 men, 77,108 women, and 23,147 children under the age of 5 years. The study area has 24 kebeles with a total of 29,610 households with an average family size of 4.14 (Bench Sheko zone health department). The study area map is designed on ArcGIS 10.5 to depict the sampled kebeles such as Kasha, Serti, Endakel, and Yali (Figure 1).

### Study design and sample population

A community-based cross-sectional study design was employed using a two-stage design to select the study participants who fulfilled the inclusion criteria.

#### Inclusion criteria

Households or caregivers who express the willingness to participate in this study will be included to provide the required information. Conversely, participants or caregivers who are unable to participate due to their busy time shall be excluded from this study.

#### Sample size calculation

The following assumptions were used to compute the sample size: due to a lack of prior similar research, a 50% proportion of the association of COVID-19 prevention practices in a rural community was used, along with a 95% confidence level and a 5% margin of error, including 10% for non-responses. A design effect of 1.82 since the study participants were recruited using a two-stage design, due to the constraint and limitations of the relevant resources such as budget and time, which must be considered for the study (7).


$$\begin{array}{l} n=\frac{\;{\left(z\alpha /2\right)}^{2}\left(1-p\right)*0.5}{d^{2}}, \end{array}$$

where, *n* = sample size, q = 1 – p, d = margin of error = 0.05(5%)


$$\begin{array}{l} n=\frac{({\left(1.96\right)}^{2}*(1.96))*0.5}{\;\;{\left(0.5\right)}^{2}})*1.82*10\%=768 \end{array}$$

Therefore, 768 total sample sizes were obtained for the study and uneven distributions of sample size were applied to select the respondents from sampled kebeles.

#### Sampling technique

A simple random sampling technique was used to choose the study participants based on eligibility criteria, ensuring that every household in a population has an equal chance of being chosen for the sample. Simple random sampling is a type of probability sampling technique in which the researcher randomly selects study participants from all households in the kebeles. Each member of the household has an equal chance of being selected for the study. The data are then collected from as large a percentage as possible of this random subset. Only one person from each family was selected to control the risk of contact with COVID-19 and to reduce the clustering effect and potential bias, specifically, the need to control for confounding variables related to family clustering.

### Data collection process and tools

The survey questionnaires were adapted from previously published COVID-19 surveys that included demographic information, 11 knowledge items, and five practice items (8–10). A pretest was conducted on 5% of the samples in Sheko kebeles (Sheko district) and necessary changes were made to tools.

To assess the knowledge about COVID-19, 11 items were included in the participant knowledge questionnaires. Items 1–5 covered clinical presentations, items 6–8 focused on transmission routes, and items 9–11 were related to prevention and control of COVID-19. A correct response to an item earned one point, while an incorrect or uncertain response earned zero points. The total score ranged from 0 to 11 with higher scores indicating good COVID-19 knowledge. Similarly, the COVID-19 preventative practice questionnaire consisted of five items with a total score ranging from 0 to 5. The classification of knowledge and practice was adapted from previous research and applied to evaluate the knowledge and practice levels of the respondents. Additionally, knowledge and practices were operationalized as the awareness levels of the respondents exceeded the mean score on questions related to knowledge and practices regarding COVID-19 (9).

### Study variables of interest

#### Outcome variable

The prevalence of childhood diarrhea within 2 weeks before the data collection is considered a dependent variable.

#### Independent variables

The independent variables included knowledge and practices of COVID-19 prevention, sociodemographic variables, and WASH facilities (critical handwashing times were identified as before preparing food, eating, providing supplementary feeding for children, breastfeeding, after defecation, and cleaning a child after defecation) (11). Per capita water consumption (l/c/d) is measured as at least 20 l of water required for a person per day (an indication of the adequacy of water), and time to fetch water is defined as the total time taken to fetch water from sources within 30 min (an indication of the accessibility of water) (12).

#### Data quality assurances

The questionnaires were written in English before being translated into Amharic. Six data collectors and two supervisors were trained to ensure the quality of data. The principal investigator kept track of the data collection process daily.

### Statistical analysis

Epidata3.1 was used to enter the data, and the data were exported to Stata 14 for analysis. Frequencies and percentages were used to summarize the studied variables. The variance inflation factor (VIF) less than 10 as a measure of the collinearity of each variable was used to examine the multicollinearity between independent variables (13, 14). A chi-squared test was performed to understand the association of COVID-19 prevention practices with the occurrence of childhood diarrhea. A binary logistic regression model was used to find the potential variables related to poor COVID-19 preventative practices and the occurrence of childhood diarrhea with a *p* < 0.25 obtained from univariate analysis. The results of the final model were interpreted using an adjusted odds ratio (AOR) with a 95 percent confidence interval at a statistical significance threshold of 0.05.

### Ethical consideration

The Institutional Review Board (IRB) of Jimma University approved the study documents (IRB000197/20) and provided an ethical letter to conduct this study. In addition, consent was obtained from each participant to proceed with the data collection process.

## Results

### Sociodemographic characteristics of participants

The sociodemographic characteristics of participants were analyzed to assess the implication of COVID-19 prevention practices on the occurrence of childhood diarrhea. A total of 720 respondents completed the survey, yielding a response rate of 93.8%. More than three-fourths (73.8%) of the study participants were female individuals. The mean age of the respondents was 29.4 years with a standard deviation (SD) of 5.19. More than half, (54.6%), of the respondents, were over 40 years old, with 550 (76.4%) being married. Less than half (48.10%) of the respondents did not attend formal education. Less than half (46.9%) of respondents were farmers, while 554 (76.9%) of the respondents had more than five family members (Table 1).

**Table 1:** Sociodemographic characteristics of study participants.

**Variable**	**Categories**	**Number**	**%**
Age (in year)	18–29	120	16.7
	30–39	207	28.8
	≥40	393	54.6
	Mean (± SD)	29.4 ± 5.54	
Sex	Male	189	26.3
	Female	531	73.8
Marital status	Single	170	23.6
	Married	550	76.4
The educational level of the caregiver	No formal education	346	48.1
	Primary education	250	34.7
	More than secondary education	124	17.2
Occupation	Farmer	338	46.9
	Merchant	80	11.1
	Student	145	20.1
	Government employee	38	5.3
	Housewife	119	16.5
Family size	≥5	166	23.1
	< 5	554	76.9

The WASH facilities available to the respondents in a rural community were evaluated in relation to COVID-19 prevention practices in the Semen Bench district, southwestern Ethiopia. Almost a quarter (24.6%) of the respondents owned hand-washing facilities. Approximately 259 (36%) respondents practiced hand washing at critical times. Only 245 (34%) households obtained water from sources within 30 min of walking distance, while 276 (38.3%) respondents consumed more than 20 l per capita per day (38.3%) (Table 2).

**Table 2:** WASH facilities of the study participants.

**WASH facilities**		**Number**	**%**
Handwashing facilities	Yes	177	24.6
	No	543	75.4
Handwashing at critical times	Yes	259	36
	No	461	64
Total time to fetch water from sources, in min	< 30	245	34
	>30	475	66
Per capita water consumption per day (l/c/d)	>20	276	38.3
	< 20	444	61.7

### Knowledge of respondents toward COVID-19

Less than half, 344 (48%), of the respondents know the mode of transmission of the pandemic. A majority of the participants, 669 (93%), know a symptom of COVID-19. In addition, dry cough and breathing difficulties were mentioned as symptoms of COVID-19 (Table 3).

**Table 3:** Knowledge of respondents toward COVID-19 prevention practices.

**Characteristics**		**Response**	
		**Yes (%)**	**No (%)**
Mode of transmission	Breathing, sneezing, cough	337 (47)	383 (53)
	Physical contacts	298 (41)	422 (59)
	Handshake	344 (48)	376 (52)
Symptoms of COVID-19	Fever	669 (93)	50 (7)
	Dry cough	378 (52.5)	342 (47.5)
	Breathing difficulty	259 (36)	461 (64)
	Fatigue	89 (12.4)	631 (87.6)

### COVID-19 prevention practices

The result revealed that less than half, 349 (48.5%), of the participants had good practices for preventing the COVID-19 pandemic. The participants with no formal education were 36% less likely to implement COVID-19 prevention practices than those with secondary education (AOR = 0.638, 95% CI: 0.421, 0.967, *P* = 0.012).

Similarly, the respondents with poor knowledge were 27.2% less likely to practice COVID-19 prevention methods than those with better knowledge levels of COVID-19 (AOR = 0.728, 95% CI: 0.542, 0.978, *P* = 0.035).

The respondents who traveled more than 30 min to fetch water from sources were 35.2% less likely to practice prevention than those who obtained water < 30 min from sources (AOR = 0.675, 95% CI: 0.465, 0.980, *P* = 0.039) (Table 4).

**Table 4:** Factors of COVID-19 prevention practices.

**Variable**	**COVID-19 prevention practice**					
	**Poor**	**Good**	**COR (95%CI)**	***P*** **-value**	**AOR (95%CI)**	***P*** **-value**
**Sex**						
Male	92	97	1			
Female	262	269	0.832 (0.597, 1.161)	0.279		
**Marital status**						
Single	99	71	1.012 (0.718,1.428)	0.994		
Married	255	295	1			
**Educational status**						
No formal education	189	157	0.638 (0.421, 0.967)	0.034	0.640 (0.421,0.974)	0.037^*^
Primary education	131	119	0.665 (0.430, 1.028)	0.065	0.678 (0.437,1.051)	0.082
More than secondary education	51	73	1			
**Occupational status**						
Farmer	155	183	0.581 (0.392, 0.862)	0.007	0.569 (0.383,0.847)	0.005^*^
Merchant	42	38	1.176 (0.562, 2.459)	0.667	1.107 (0.527,2.327)	0.787
Student	86	59	0.798 (0.489, 1.302)	0.367	0.762 (0.465, 1.248)	0.281
Housewife	64	55	0.758 (0.438, 1.314)	0.607	0.753 (0.433, 1.309)	0.314
Government employee	24	14	1			
**Family size**						
≥5	85	81	0.983 (0.695, 1.391)	0.695		
< 5	286	268	1			
**Knowledge score**						
Poor	198	181	0.579 (0.431, 0.778)	0.033	0.728 (0.542,0.978)	0.035^*^
Good	190	151	1			
**Handwashing facilities**						
Yes	60	117	1			
No	311	232	0.614 (0.436, 0.864)	0.005	0.621 (0.440, 0.875)	0.007^*^
**Per capita water consumption**						
Greater than 20	160	224	1			
Less 20	211	125	0.423 (0.313, 0.571)	< 0.001	0.446 (0.329, 0.605)	0.001^*^
**Time travel to fetch water from sources**						
Greater than 30	251	143	0.516 (0.381, 0.698)	< 0.001	0.675 (0.465, 0.980)	0.039^*^
Less 30	120	206	1			
**Handwashing at critical times**						
Yes	107	153	1			
No	264	196	0.699 (0.514, 0.949)	0.022	0.705 (0.519, 0.958)	0.025^*^

^*^Significant at p < 0.05; COR, Crude odds ratio; AOR, Adjusted odds ratio; CI, confidence. Interval.

### Implication of COVID-19 prevention practices on occurrences of childhood diarrhea

This study explores the potential impacts of COVID-19 prevention measures, such as hand hygiene and increased sanitation practices, on the incidence and severity of childhood diarrhea. We can gain insights into how these prevention practices can contribute to reducing the burden of childhood diarrhea. Understanding the implications of COVID-19 prevention practices on occurrences of childhood diarrhea is crucial for developing effective strategies for disease prevention and management. By examining the reported associations between health-seeking behaviors, such as the adoption of a healthier lifestyle and good personal hygiene practices, we can assess the potential impact of these practices on reducing the transmission of diarrheal diseases among children during the COVID-19 pandemic.

The highest incidence of childhood diarrhea was observed among respondents with poor knowledge and practices regarding COVID-19 prevention. However, respondents with good knowledge of COVID-19 had a lower prevalence (7.92%) of childhood diarrhea than those with poor COVID-19 prevention knowledge (11.4%). Similarly, lower (7.22%) risks of developing childhood diarrhea were significantly associated with good preventive practices for COVID-19.

Moreover, the results from the multivariable analysis revealed that respondents with poor prevention knowledge of COVID-19 were 42% (AOR = 0.580, 95% CI: 0.398, 0.847, *P* = 0.005) less likely to develop childhood diarrhea than those who had good knowledge of COVID-19 prevention. Similarly, households with poor preventive practices for COVID-19 were 75.1% more likely to develop childhood diarrhea than those who had good preventive practices for COVID-19 (AOR = 1.751, 95% CI: 1.193, 2.571, *P* = 0.004). There is an observed significant positive association between COVID-19 prevention practices and a lower risk of childhood diarrhea (p-value less than 5) (Table 5).

**Table 5:** Implication of COVID-19 prevention practices on occurrences of childhood diarrhea.

**COVID-19 prevention**		**Childhood diarrhea within two weeks**					
		**Yes (%)**	**No (%)**	**COR (95% CI)**	***p*** **-value**	**AOR (95% CI)**	***p*** **-value**
Knowledge	Poor	82 (11.40)	259 (36)	0.559 (0.384, 0.814)	0.002	0.580 (0.398, 0.847)	0.005^*^
	Good	57 (7.92)	322 (44.72)	1			
Practices	Poor	87 (12.10)	267 (37.10)	1.817 (1.241, 2.661)	0.002	1.751 (1.193, 2.571)	0.004^*^
	Good	52 (7.22)	314 (43.61)	1			

^*^Significant at p < 0.05; COR, Crude odds ratio; AOR, Adjusted odds ratio; CI, confidence interval.

## Discussion

This study showed that good COVID-19 prevention practices reduced the prevalence of childhood diarrhea compared to groups with poorly practiced respondents. The previous findings suggested that COVID-19 intervention, particularly proper handwashing practices, could reduce episodes of diarrhea (15, 16). Moreover, the COVID-19 prevention practices of frequent hand washing with soap and sanitizers were also the existing key NPIs for preventing acute childhood diarrhea globally. These practices, promoted for COVID-19 prevention, provide a valuable opportunity to explore the implication of COVID-19 prevention practices on the occurrence of childhood diarrhea.

The prevalence of childhood diarrhea during the research period was 19.3%, which is lower than the previously reported prevalence from similar contexts before the pandemic (17). The lower prevalence of childhood diarrhea in the current study might suggest some behaviors, such as handwashing with soap, may have improved as a result of promotions of frequent and proper handwashing practices during the pandemic (17). Proper hand hygiene reduces the chances of ingesting or coming into contact with pathogens, leading to a decrease in diarrhea cases. However, the COVID-19 pandemic has increased the demand for water and sanitation worldwide, highlighting the need for basic WASH in both households and public places (18).

This study indicated that more than half of the respondents (55.3%) were aware of COVID-19 prevention methods, a finding that is similar to a study conducted in Bangladesh (54.87%) (19) but inconsistent with findings from Malaysia (80.5%) (20) and India (80.64%) (21). The limited COVID-19 prevention awareness in this study was influenced by the respondent's educational status and rural residency. These two factors affected their access to various social media platforms through which health information was disseminated. Several findings revealed that educated respondents had more access to various information sources on COVID-19 (21–23) to prevent the pandemic. This access may increase the likelihood of being more aware of the spread of the pandemic, enabling better implementation of recommended measures and potentially reducing the incidence of childhood diarrhea (24).

In this study, the COVID-19 prevention practice was less than half, 48.5%, which is inconsistent with the studies conducted in India (83.8%) (25), southern Ethiopia (80%) (26), and Saudi Arabia (81%) (27). The poor practices of COVID-19 prevention in this study area were affected by the poor knowledge level, absence of formal education, and WASH facilities (Table 5), which is similar to the earlier suggested factors (1). In developing countries, pre-existing WASH facilities were key intervention strategies against various infections. Currently, there are NPIs that control the spread of the COVID-19 pandemic (28); however, the coverage of WASH facilities in this study area needs due attention from the health sectors. The findings on prevention practices might be dissimilar because this study was conducted in rural communities with limited access to health information and a lower level of educational status compared to the participants in other studies. In addition, the mean score taken as a reference to distinguish between good and poor prevention practices of COVID-19 and the sample size might also contribute to the difference.

This study has provided evidence of the implications of COVID-19 prevention practices in relation to occurrences of childhood diarrhea that could aid health implementers, planners, and policymakers in integrating strategies and generating hypotheses.

### Limitations of the study

Since this study used simple random sampling techniques to select a sample, sampling errors may have occurred. The sample may not accurately reflect the general population or the appropriate population of interest which may cause “sample bias” or “selection bias”. This occurs when a study systematically differs from the population of interest resulting in a systematic error in the association or outcome. However, the chi-squared test was used to measure associations or implications that may not address the cause–effect relationship. Further study using prospective study designs with advanced statistical approaches is needed.

In this study, biases were managed by carefully utilizing simple random sample techniques, pretesting and translating data collection tools. The adjusted odds ratio analysis was also used to control for any confounding variables.

## Conclusion

COVID-19 prevention practices were significantly associated with a lower risk of developing childhood diarrhea. The increasing level of knowledge is crucial for preventing the COVID-19 pandemic, which has consequently helped reduce the risk of childhood diarrhea.

## Data availability statement

The raw data supporting the conclusions of this article will be made available by the authors, without undue reservation.

## Ethics statement

The studies involving humans were approved by the Institutional Review Board (IRB) of Jimma University (IRB000197/20). The studies were conducted in accordance with the local legislation and institutional requirements. The participants provided their written informed consent to participate in this study.

## Author contributions

BA: Conceptualization, Data curation, Formal Analysis, Funding acquisition, Investigation, Methodology, Project administration, Resources, Software, Supervision, Validation, Visualization, Writing—original draft, Writing—review & editing. SM: Conceptualization, Data curation, Formal analysis, Funding acquisition, Investigation, Methodology, Project administration, Resources, Software, Supervision, Validation, Visualization, Writing—review & editing. AA: Conceptualization, Data curation, Formal analysis, Funding acquisition, Investigation, Methodology, Project administration, Resources, Software, Supervision, Validation, Visualization, Writing—review & editing.
